# An ancient polymorphic regulatory region within the BDNF gene associated with obesity modulates anxiety-like behaviour in mice and humans

**DOI:** 10.1038/s41380-023-02359-7

**Published:** 2024-01-16

**Authors:** Andrew R. McEwan, Benjamin Hing, Johanna C. Erickson, Greg Hutchings, Charity Urama, Emily Norton-Hughes, Mariam D’Ippolito, Susan Berry, Mirela Delibegovic, Felix Grassmann, Alasdair MacKenzie

**Affiliations:** 1 School of Medicine, Medical Sciences and Nutrition, Institute of Medical Sciences, University of Aberdeen, Foresterhill, Aberdeen, AB24 2ZD UK; 2 Department of Psychiatry, Carver College of Medicine, University of Iowa, Iowa City, IA USA; 3 Institute for Clinical Research and Systems Medicine, Health and Medical University, Potsdam, Germany

**Keywords:** Genetics, Depression

## Abstract

Obesity and anxiety are morbidities notable for their increased impact on society during the recent COVID-19 pandemic. Understanding the mechanisms governing susceptibility to these conditions will increase our quality of life and resilience to future pandemics. In the current study, we explored the function of a highly conserved regulatory region (BE5.1) within the BDNF gene that harbours a polymorphism strongly associated with obesity (rs10767664; *p* = 4.69 × 10^–26^). Analysis in primary cells suggested that the major T-allele of BE5.1 was an enhancer, whereas the obesity-associated A-allele was not. However, CRISPR/CAS9 deletion of BE5.1 from the mouse genome (BE5.1KO) produced no significant effect on the expression of BDNF transcripts in the hypothalamus, no change in weight gain after 28 days and only a marginally significant increase in food intake. Nevertheless, transcripts were significantly increased in the amygdala of female mice and elevated zero maze and marble-burying tests demonstrated a significant increase in anxiety-like behaviour that could be reversed by diazepam. Consistent with these observations, human GWAS cohort analysis demonstrated a significant association between rs10767664 and anxiousness in human populations. Intriguingly, interrogation of the human GTEx eQTL database demonstrated no effect on BDNF mRNA levels associated with rs10767664 but a highly significant effect on BDNF-antisense (BDNF-AS) gene expression and splicing. The subsequent observation that deletion of BE5.1 also significantly reduced BDNF-AS expression in mice suggests a novel mechanism in the regulation of BDNF expression common to mice and humans, which contributes to the modulation of mood and anxiety in both species.

## Introduction

The recent COVID-19 pandemic has highlighted the effects of obesity on COVID-19 mortality and the increased levels of anxiety suffered worldwide as a result of the lockdown [1]. Because of differing levels of susceptibility to the effects of COVID-19 in different populations, a great deal of interest has focussed on the mechanisms regulating susceptibility to obesity and anxiety and the effects of genetic variation. The gene encoding the brain-derived neurotrophic factor (BDNF) is particularly relevant in this regard as genome-wide association study (GWAS) associated polymorphisms around the BDNF gene locus have been associated with conditions including depression [2], anxiety [3] and obesity [4, 5]. However, the vast majority of disease-associated single-nucleotide polymorphisms (SNPs) in and around the BDNF gene locus are non-coding. This observation is consistent with the results of thousands of other GWAS studies and suggests that altered cell-specific regulation of BDNF gene expression is an important factor in disease susceptibility [6]. Because of its importance in brain development and function, and its association with multiple diseases, much has been learned about the regulation of the BDNF gene [2, 3]. However, these studies have only scratched the surface of the complex regulation of this gene and much more work remains to be done, not only to discover the functional elements involved, but also the effects of allelic variation at the cell-specific level.

The BDNF gene encodes a secreted protein that binds the TrkB receptor where it helps to support the survival of existing neurons, encourages growth and differentiation of new neurons and synapses, and is important in neuronal plasticity [7]. The gene encoding the BDNF protein has 9 exons, each with its own promoter [8]. Despite the great complexity of the regulation of its expression, only exon 9 encodes the BDNF protein so the fact that the gene is controlled by 9 different promoters that produce 17 different transcripts, speaks to the high level of transcriptional control required for proper cell-specific expression of the gene in the healthy brain [8]. Diverse BDNF transcripts, produced from different promoters, serve distinct molecular and behavioural functions including aggressiveness and modulation of components of the serotoninergic system that plays a role in mood modulation [9] and appetite [10]. One of the promoters driving the expression of BDNF; promoter IV (BP4), was found to play a role in anxiety and depression-like behaviour when deleted from mice [11]. There is also evidence that methylation of BP4 plays an important role in resistance to the effects of antidepressant therapies [12]. In addition to promoter regions [13], studies have also characterised many of the enhancer and repressor regions that support the tissue-specific expression of BDNF [14, 15]. Efforts to identify enhancers that control BDNF have employed high throughput next-generation sequencing (NGS) technologies such as ATAC-seq [16] and chromatin immunoprecipitation sequencing (ChIP-seq) to detect markers of active enhancers (H3K4me1, H3K27Ac) [17]. These technologies have allowed the successful identification of an enhancer region (+3 kb enhancer) within the rat BDNF intron 3 [15]. The same group were also successful in identifying a role for the CREB family of transcription factors in regulating BDNF and described an autoregulatory mechanism in BDNF regulation [18]. Other efforts to identify BDNF regulatory regions combined genetic association analysis with comparative genomics to explore the effects of a polymorphism (rs12273363) associated with major depressive disorder (MDD) [11] on BDNF promoter IV activity [14]. This study found that different alleles of rs12273363 changed its ability to modulate the effects of different signal transduction pathways on BDNF promotor IV activity (BP4) [14]. It is therefore likely that combining both approaches; biochemical marker analysis and comparative genomics, with genome-wide association analysis and expression quantitative trait locus analysis, will be an effective combination to understand the tissue-specific regulation of the BDNF gene and how its misregulation, due to allelic variation, may contribute to obesity and/or pathological anxiety.

In the current study, we explored the functional biology of a SNP that had the fifth highest association to obesity (rs10767664; *p* = 4.69 × 10^−26^) in a study of 204,158 individuals [5] and which also occurred in a highly conserved region (BE5.1) within intron 3 of the BDNF gene. Based on these observations, we hypothesised that this conserved region played a role in either appetite or metabolism. We, therefore, isolated BE5.1 from the human genome and reproduced both alleles of the rs10767664 polymorphism. We then analysed the effects of these allelic variants on the activity of BP4 in reporter construct transformed into primary cell lines. In addition, CRISPR genome editing was used to delete this region from the mouse genome and the effects of this deletion on BDNF gene expression and behaviour were analysed. We finally conducted human cohort association analyses and eQTL analysis of rs10767664 allelic variants. The intriguing results of these experiments are discussed in the context of understanding the role of BDNF gene regulation in mental health and gives important insights into attempts to derive functional data from existing GWAS association analyses.

## Materials and methods

### Plasmid construction

Human BE5.1 was amplified using high fidelity PCR (Expand high fidelity system, Roche, UK) from human DNA (Cambio, UK) using the following primers; BE5.1 for. 5’-AATGAGGGAAAGTTTCACAGC-3’ and BE5.1 rev. 5’-CTGTGCCACTCTGCTCAAC-3’. Products were ligated into pGEM-T easy cloning vector (Promega, UK). All amplified DNA samples were sequenced (Source Bioscience, UK) to ensure correct sequence and orientation. To reproduce the A- variant the following primers were used for SDM by site-directed mutagenesis using QuickChange II site-directed mutagenesis kit (Agilent Technologies, UK) using the following primers; forward primer: 5’-GTAGGCTTGACATTGACATGTTTTTACTATTAATAATTTTAATTGGCTGAG-3’ and reverse primer 5’-CTCAGCCAATTAAAATTATTAATAGTAAAAACATGTCAATGTCAAGCCTAC-3’. BE5.1(A) and BE5.1(T) were then ligated into the BP4 luciferase reporter construct (based on pGL4-23) as previously described [14].

### Primary cell culture

Hypothalamic tissues were dissected from postnatal day 0–3 Sprague Dawley rat pups that were humanely sacrificed according to UK Home Office guidelines. Tissues were treated with 0.05% trypsin EDTA (Invitrogen, UK) for 15 min at 37 °C. Trypsin EDTA was replaced with soybean trypsin inhibitor (Sigma, UK) for 5 min at room temperature to stop reaction. This was then replaced with unsupplemented Neurobasal A (Invitrogen, UK) followed by mechanical dissociation. Cells were then resuspended in culture media [Neurobasal A, B27 (Invitrogen, UK), 1X glutamax (Invitrogen, UK) and Pen/strep (100 U/ml) (Invitrogen, UK)] and plated out at a density of ~80,000 viable cells/cm^2^ on poly-L-lysine (20 µg/ml) (Sigma, UK) pretreated plates following cell counting for viable cells using TC10^TM^ automated cell counter (Bio-Rad) with trypan blue. Cells were incubated at 37 °C, 5% CO_2_ for 7 days prior to transfection.

### Transfections and treatments

All DNA constructs were quantified on a nanodrop machine (NanoDrop Technologies). Quantities of plasmid used for each transfection were adjusted to the size of each plasmid to ensure molar equivalence between experiments. Firefly luciferase plasmids were co-transfected with Renilla luciferase plasmid, pGL4.70 (Promega, UK), to normalise signals between transfections using magnetic particles (Neuromag; Oz Bioscience, UK) as described in manufacturer’s instructions. Cultures were incubated for 24 h prior to harvest for dual luciferase assay as per the manufacturer’s instructions (Promega).

### Generation of gRNA molecules by a novel annealed oligo template (AOT) method

Single-guide RNA (sgRNA) molecules were designed to disrupt the mouse BE5.1 using the optimised CRISPR design tool (http://CRISPR.mit.edu/). sgRNA template was produced by annealing oligonucleotides to produce two different DNA templates using the annealed oligo template (AOT) method as previously described [19] that included a T7 polymerase binding site and predicted guide sequence target sites spanning the conserved region surrounding the mouse BE5.1 enhancer (5’sgRNA; GGCCACATCTAAGTATAGCA (chr2:109,689,202–109,689,221) and 3’sgRNA; ATCAATGGCCTGTGCATTAT (chr2:109,690,239–109,690,258). These oligonucleotides were annealed and amplified using PCR to produce a 122-bp double-strand sgRNA template. 100 ng of this template was used to produce sgRNA using an mMESSAGE mMACHINE T7 in vitro transcription kit (Ambion) described in the manufacturer’s instructions and purified using a Megaclear kit (Ambion) with modifications as previously described [20].

### Production of genome-edited mice

sgRNA molecules were microinjected at a concentration 10 ng/µl each into the cytoplasm of 1-cell C57/BL6 embryos as described [20] together with 10 ng/µl CAS9 mRNA (Life Technologies). Two-cell embryos were introduced into host CD1 mothers using oviduct transfer as previously described [21] and correctly targeted offspring were determined by PCR of earclip DNA using the following flanking primers (AMX013; GCATCCTTTTGAGAGAAAT, AMX014; CCGAGGACACAGGAAGC).

### Examination of possible off-target sites

Likely off-target sites for both of the sgRNA used to delete BE5.1 were identified using the CRISPOR web tool (http://crispor.tefor.net/) (Supplementary Table S1). PCR primers were designed (Supplementary Table S2) to amplify 200–300 base pair fragments surrounding each of the five most likely off-target sites in the mouse genome from earclip DNA derived from BE5.1KO mice using PCR. PCR products were Sanger sequenced as previously described [22]. Sequences were then submitted to the BLAT facility of the UCSC browser (Mouse BLAT Search (ucsc.edu)) and compared to the mouse genome sequence (Mouse mm39; Supplementary Fig. S2).

### Quantitative reverse transcriptase-PCR

Brain tissues (hypothalamus and amygdala) were recovered from wild-type and BE5.1KO offspring and snap frozen on dry ice. Total RNA was extracted using the isolate II RNA minikit (Bioline). Quantitative reverse transcriptase-PCR (QrtPCR) on derived mouse cDNA was carried out using BDNF splice isoform-specific primers (see Supplementary Table S3) to determine differential changes in BDNF splice forms using a Roche Light Cycler 480 with Roche SYBR green [23, 24] normalised against the Non-POU Domain Containing Octamer Binding protein (*Nono)* housekeeping gene as previously described [19, 25–27].

### Animal studies

All animal studies were performed in accordance with UK Home Office guidelines and maintained under specific pathogen-free (SPF) facilities. The health status of these animals conformed to The Federation of European Laboratory Animal Science Associations (FELASA) guidelines whereby pathogen screening was carried out on a quarterly (3 monthly) basis using sentinel animals. BE5.1KO mice were maintained as a colony on a heterozygous C57BL/6 background which were mated to produce homozygous wild-type and homozygous BE5.1KO age-matched and sex-matched individuals. These were identified using PCR of earclip DNA as described above. Once identified, animals were assigned random numbers to hide their genotype from the operators of subsequent tests. Male and female homozygous wild-type and BE5.1KO age-matched littermates were housed under standard laboratory conditions (12 h light/12 h dark cycle), in plastic cages with food and water available ad libitum, depending on the experiment.

### Food intake studies

For food intake and high-fat diet preference studies, singly housed animals were provided with a choice of standard CHOW diet (low-fat diet, LFD; 22.03 kcal% protein, 68.9 kcal% carbohydrate and 9.08 kcal% fat) or high-fat diet (HFD; 20 kcal% protein, 20 kcal% carbohydrate and 60 kcal % fat; Research Diets Inc.) in different hoppers. The position of each hopper was changed regularly to rule out the possibility of position effects. Animals were weighed daily over a period of 23 days and LFD and HFD were also weighed daily to determine intake of each diet. Animals were also subjected to ECHO MRI analysis at the start and at the end of the feeding trial to assess changes in body fat mass vs. total body mass.

### Elevated zero maze (EZM)

The EZM consists of an annular dark-grey platform (60 cm in diameter) constructed of opaque Perspex divided into four equal quadrants. Two opposite quadrants were ‘open’; the remaining two ‘closed’ quadrants were surrounded by 16 cm high dark, opaque black walls. Quadrant lanes were 5 cm in width. Overhead lighting applying 100 lx at the level of the maze. The movement of animals was tracked using a camera and ANY-maze tracking software (Stoelting Europe). Distance travelled, average speed, number of crossings between light/dark zones and freezing episodes, freezing time and freezing latency were recorded.

### Marble-burying test (MBT)

The MBT consisted of a ‘EUROSTANDARD TYPE IV S’ (Techiplast) cage (480 × 375 × 210 mm) cage with 5 cm depth of wood chip bedding onto which 20 evenly spaced marbles were placed. Animals were then placed in the cage and the numbers of marbles buried after 10 min were recorded. This experiment was repeated with the same animals following i.p. injection of 10 mg/kg of diazepam.

### Assessment of rs10767664 in human populations

We extracted the association of rs10767664 with all traits available in the MRC IEU OpenGWAS database [https://europepmc.org/article/ppr/ppr199104] with the function *phewas* from the *ieugwasr* package [Gibran Hemani (2021). ieugwasr: R interface to the IEU GWAS database API. R package version 0.1.5. URL: https://github.com/mrcieu/ieugwasr], as implemented in R [R Core Team (2020). R: A language and environment for statistical computing. R Foundation for Statistical Computing, Vienna, Austria. URL: https://www.R-project.org/]. We restricted the results to all associations related to anxiety, worry, risk taking and sexual history. To account for multiple testing, we controlled the false-discovery rate to be less than 5% and thus report all associations with a Q-value of less than 5%. eQTL analysis of the effects of rs10767664 on human gene expression was undertaken by interrogation of the GTEx Portal (https://www.gtexportal.org/home/).

### Data analysis

From in vivo pilot studies, we calculated that a minimum of 6–12 animals per group would enable the detection of a 25% difference between different parameters (anxiety-like behaviour, food intake) with 80% power using one-way ANOVA and/or general linear modelling. Statistical significance of datasets was analysed using either one-way analysis of variance (ANOVA) analysis with Bonferroni post hoc tests or using two-tailed unpaired parametric Student *t*-test as indicated using GraphPad PRISM version 5.02 (GraphPad Software, La Jolla, CA, USA).

## Results

### The obesity-associated polymorphism rs10767664 occurs within intron 3 of the BDNF gene and within a sequence highly conserved between humans, mice and amphibians

**Fig. 1:**
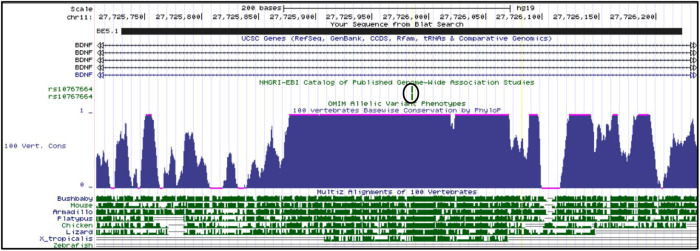
The rs10767644 polymorphisms occurs within a region of the genome conserved for 350 million year. UCSC browser images covering 500 base pairs of the human BDNF intron 3, demonstrating relative conservation (blue peaks and pink lines) and depth of conservation through evolution (green bars and lines, *X. tropicalis* = 350 million years divergence) that hosts the obesity GWAS associated rs10767664 polymorphism (circled in black).

Because of its importance in obesity [5] and the importance of the BDNF gene in appetite control [28], we explored the hypothesis that allelic variation at the rs10767664 locus functionally changed the activity of an uncharacterised cis-regulatory element that controls aspects of the expression of the BDNF gene involved in appetite. We were intrigued to find that rs10767664 occurred within a 494 base pair region of highly conserved DNA (BE5.1; chr11:27,725,730–27,726,223), specifically within a 240-bp region of the extreme conservation that demonstrated >90% conservation to amphibians; a depth of conservation spanning 360 million years of divergent evolution [29] (Fig. 1). BE5.1 lay 2.4 kb 5’ from promoter IV of the BDNF gene (BP4), within BDNF intron 3, (Supplementary Fig. S1) and 20 kb away from the recently reported +3 kb enhancer [15].

### The T-allele of BE5.1 acts as an enhancer of BP4 activity in primary hypothalamic cells

**Fig. 2:**
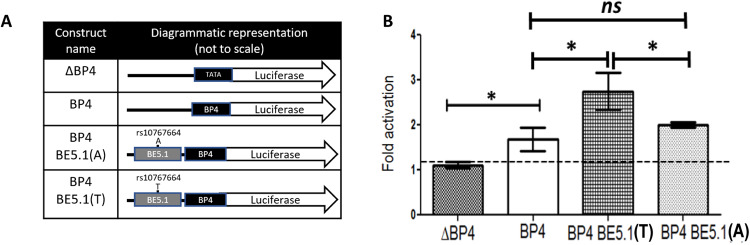
The rs10767664 polymorphism alters BE5.1 enhancer activity. **A** Diagrammatic representation of the construct used in (**B**). Column 1 indicates the construct names used in (**B**). Column 2, a diagrammatic representation showing the relationship of each of the components. TATA; minimum TATA box promoter, BP4; BDNF promoter 4, BE5.1; BDNF enhancer 5.1. **B** Bar graphs showing the firefly luciferase activities of each construct in (**A**), 48 h by dual luciferase analysis after magnetofection into primary hypothalamic cells (normalised to a co-transfected renilla luciferase constructs). Activity is expressed as fold activity compared to the minimal TATA box promoter construct (ΔBP4). *p < 0.05, ns; not significant, error bars = SEM.

To determine the effects of different alleles of BE5.1 on BDNF promoter activity we cloned 494 bp surrounding the human BE5.1 sequence using Hi-fidelity PCR and reproduced both human alleles using site-directed mutagenesis. Both allelic variants were then cloned into a luciferase reporter plasmid containing the BP4 promoter region (Fig. 2A), which represented the closest promoter to BE5.1, as previously described [14]. We also chose this promoter as BP4 had previously been shown to be active in hypothalamus and amygdala in transgenic animals [14]. These reporter plasmids were transfected into primary rat hypothalamic cells by magnetofection as previously described [14] and incubated for 48 h. After this time, cells were lysed and lysates were analysed by dual luciferase assay. In this way, we were able to demonstrate that the T- allele of BE5.1 could act as an enhancer of BP4 activity but that the obesity-associated A-allele could not (Fig. 2B).

### BE5.1 knockout animals are healthy and viable

We used CAS9/CRISPR genome editing to delete the BE5.1 enhancer from the mouse genome by cytoplasmic injection of single-guide RNA (sgRNA) and CAS9 mRNA into 1-cell C57BL/6 mouse embryos as previously described [19, 25–27]. We were able to generate a single heterozygous female animal (BE5.1KO^+/-^) which was outbred on a C57BL/6 background for two generations to produce a colony of heterozygous male and female BE5.1 KO animals. Sequence analysis of the 5 most likely off-target events for each sgRNA (Supplementary Tables S1 and S2) was carried out as previously described [22] and we were unable to detect any evidence of off-target changes within the genome of this line (Supplementary Fig. S2). These animals were then bred together to produce homozygous wild-type (WT) and BE5.1KO animals, selected on the basis of PCR analysis, which proved to be healthy and viable (Supplementary Table S5).

### Deletion of BE5.1 has no significant effect on expression of BDNF isoforms in the hypothalamus

**Fig. 3:**
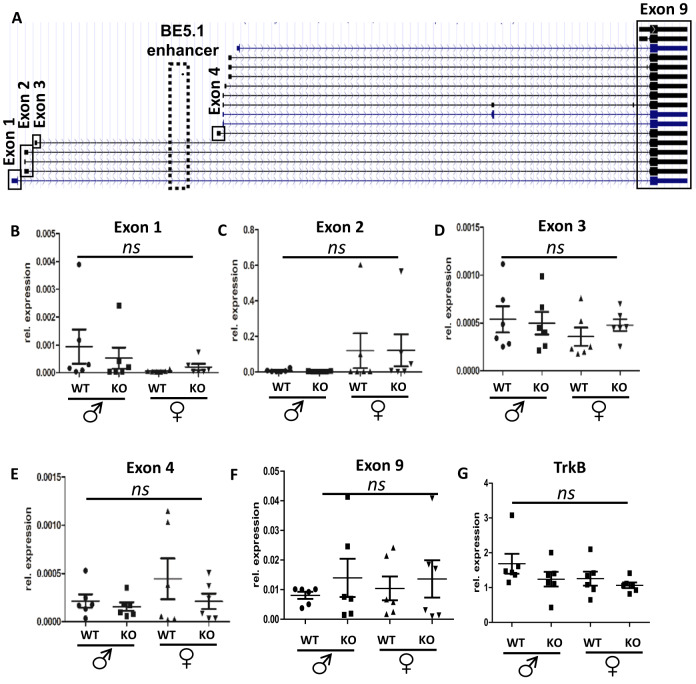
Deletion of BE5.1 does not significantly affect BDNF mRNA expression in hypothalamus. **A** Figure derived from UCSC browser showing the different known BDNF mRNA isoforms and the spatial relationships between each promoter (black boxes) and the relative location of the BE5.1 enhancer (broken box). **B**–**G** Results of QPCR analysis of BDNF and TRKB mRNA derived from the hypothalamus of wild-type (WT) and BE5.1KO (KO) male and female mice comparing levels of BDNF mRNA isoform expression from promoters 1–4 and 9 (exons 1–4 and 9) between WT and BE5.1KO male and female animals. ns, not significant, error bars = SEM.

To determine the possible effects of the BE5.1 deletion on the expression of BDNF mRNA splice forms at a tissue-specific level, we recovered total RNA from the hypothalamus of male and female WT and BE5.1KO littermates. The hypothalamus was initially chosen as this region of the brain is responsible for appetite control and changes in the expression of BDNF in hypothalamus affect food intake [30]. We then used quantitative reverse transcriptase-PCR (QPCR) to quantify the expression of different isoforms of BDNF derived from different promoters (Fig. 3A and Supplementary Table S3) in the hypothalamus where expression of BDNF influences appetite [31]. We focused on exons 1–5 because these exons are driven by promoters known to modulate the expression of BDNF involved in appetite control and mood [9, 10]. We also explored the expression of exon 9 which contained the entire coding region of BDNF. However, we were unable to detect any significant difference in the expression of BDNF isoforms expressed from promoters 1–5 (exon 1–5) or promoter 9 (exon 9) in these samples (Fig. 3B–F). In order to determine the effects of deleting BE5.1 on the expression of the receptor for BDNF we also undertook QPCR analysis of the TrkB receptor but found no change in its expression either (Fig. 3G).

### Deletion of the BE5.1 enhancer had no significant effect on weight gain and only marginal effects on food intake

**Fig. 4:**
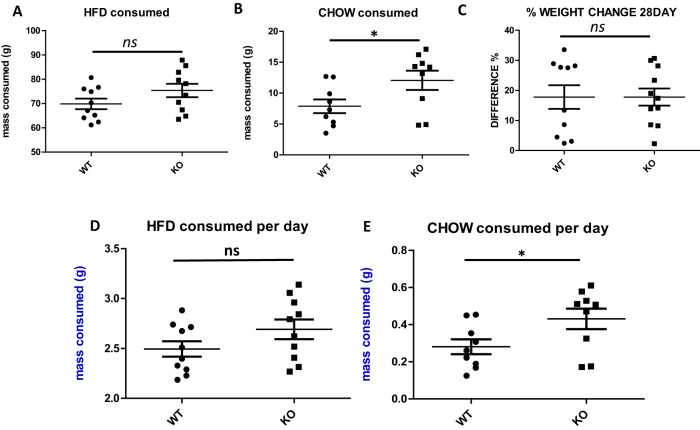
Deletion of BE5.1 does not significantly affect food intake or weight gain. Scatter plots demonstrating the total mass of **A** high-fat diet and **B** low-fat diet (CHOW) consumed either over the 28 days of the study (or **D** and **E** per day), comparing wild-type (WT) and BE5.1KO (KO) animals. **C** percentage change in total body mass comparing WT and KO animals exposed to a choice of high-fat and CHOW diet over 28 days. ns, not significant; *p < 0.05; error bars = SEM.

Because of the association of the rs10767664 polymorphism within BE5.1 with obesity, we explored the hypothesis that deletion of BE5.1 from the mouse genome would produce a significant change in food intake or weight gain. We allowed WT and BE5.1KO littermates access to a choice of either standard CHOW or high-fat diet (HFD) for 28 days. Animals were weighed at regular intervals and analysed at the beginning and end of 28 days using echo MRI analysis. Although we detected a marginally significant increase in the intake of CHOW diet in BE5.1KO animals (Fig. 4B, E), consistent with previously described effects of decreased BDNF expression [28], we could not identify any significant differences in the intake of high-fat diet, weight gain or fat distribution in these animals after 28 days suggesting that BE5.1 had only marginally significant effects on food intake and no significant effect on weight gain over the period of time studied (Fig. 4A, C, D).

### Deletion of BE5.1 significantly up-regulates expression of BDNF exons in the female amygdala

**Fig. 5:**
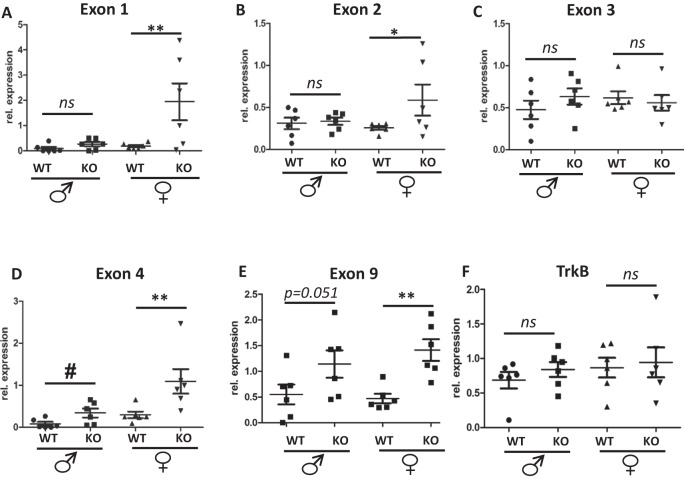
Deletion of BE5.1 increases BDNF mRNA in female amygdala. **A**–**F** Results of QPCR analysis of different exons of BDNF (**A**–**E**) and TRKB (**F**) mRNA derived from the amygdala region of wild-type (WT) and BE5.1KO (KO) male and female mice comparing levels of mRNA expression between WT and BE5.1KO male and female animals normalised against Nono gene expression. ns, not significant; *p < 0.05, **p < 0.01 (by ANOVA); ^#^*p* < 0.05 (two-tailed *t*-test).

We also recovered tissues from the amygdala of male and female WT and BE5.1KO littermates and used QPCR to quantify the expression of different exons of BDNF in the amygdala where the expression of BDNF is known to influence anxiety-like behaviour [32]. Although we observed a trend in the data towards increased expression of BDNF exons 4 and 9 in these samples in males the increase was only marginally significant for exon 4 (by *t*-test) and fell just short of significant for exon 9 (*p* = 0.051) (Fig. 5A–E). However, compared to WT littermates we detected a significant increase in the expression of exons 1, 2 and 4, (excluding exon 3), and exon 9 in amygdala tissues derived from BE5.1KO female mice (Fig. 5A–E).

### Female BE5.1KO mice demonstrate increased anxiety-like behaviour in the elevated zero maze

**Fig. 6:**
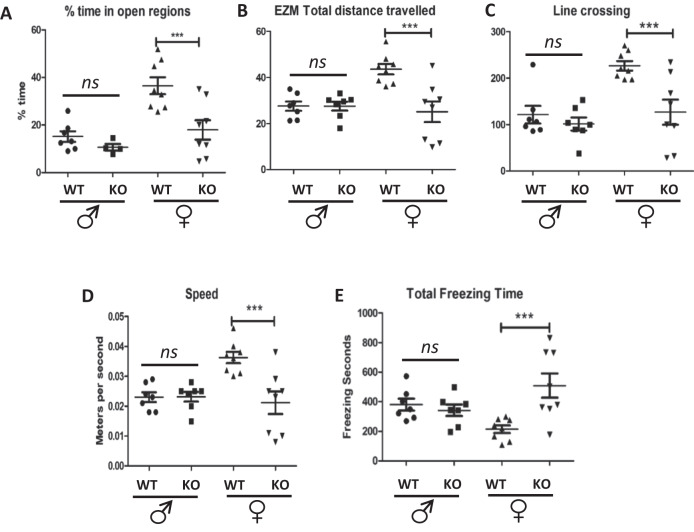
Deletion of BE5.1 increases female anxiety-like behaviours in the EZM. **A**–**E** Scatterplots demonstrating the effects of deleting BE5.1 on the behaviour of wild-type (WT) and BE5.1KO (KO) animals subjected to the elevated zero maze demonstrating **A** percentage time that each animal spent in the open quadrants of the maze, **B** the total distance travelled, **C** the number of crossings between the covered and open quadrants, **D** the average speed of each animal during the test and **E** the total time spent immobile (freezing behaviour). ns, not significant; *p < 0.01; ***p < 0.005.

Because we detected increased levels of 4 different BDNF exons in the amygdala of female BE5.1KO mice we asked whether deletion of BE5.1 would influence anxiety-like behaviour in these animals. We subjected male and female BE5.1KO and WT littermates to the elevated zero maze (EZM) which is a robust method of detecting anxiety-like behaviour in mice [33]. However, we were unable to detect any significant difference between the time spent in the open quadrants (Fig. 6A), total distance travelled (Fig. 6B), Line crossings (Fig. 6C) speed (Fig. 6D) or freezing time (Fig. 6E) between male WT or BE5.1KO littermates in the EZM. By contrast, we observed highly significant changes in all of these behaviours in female BE5,1KO mice who spent significantly less time in the open quadrants of the EZM, showed reduced line crossings and overall speed with a highly significant increase in freezing time that accounted for much of the perceived lack of mobility in these animals (Fig. 6A–E).

### Deletion of BE5.1 decreases marble burying in female mice

**Fig. 7:**
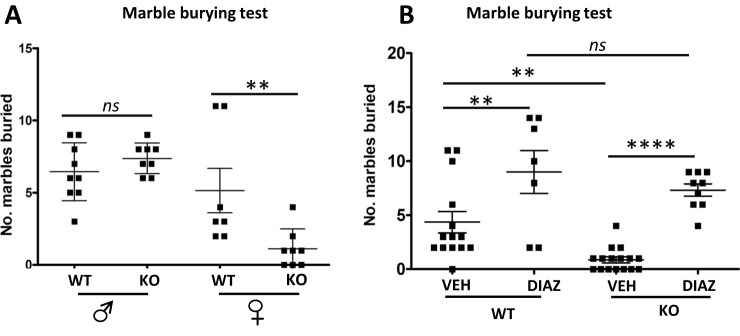
Deletion of BE5.1 increases female anxiety-like behaviours in the MBT. **A** Scatterplots demonstrating the effects of deleting BE5.1 on marble-burying behaviour in wild-type (WT) and BE5.1KO (KO) animals. **B** The effects of diazepam (DIAZ; 10 mg/kg^–1^ I.P.) on marble burying in female WT and BE5.1KO mice. ns, not significant; **p < 0.01; ****p < 0.005; error bars = SEM.

In order to further test the effects of BE5.1 deletion on anxiety-like behaviour in mice, we exposed male and female WT and BE5.1KO animals to a second anxiety test called the marble-burying test (MBT), a well-characterised test of anxiety-like behaviour in rodents [34]. Mice habitually bury marbles placed in their environment; a behaviour which decreases with increased anxiety. The advantage of the MBT is that, unlike the EZM; where animals become habituated to the test, the MBT can be repeated many times with the same animals with minimal change in the numbers of marbles each animal buries [34]. Briefly, the animals were placed in a cage with 3 cm depth of fresh bedding (wood shavings) onto which 20 × 1.5 cm glass marbles were placed. After 30 min, the animals were removed from the cage and the number of marbles buried was recorded. In the case of male animals, no significant difference was observed in the number of marbles buried by either the wild-type or the BE5.1KO animals where both groups buried and average of 6–7 marbles each (Fig. 7A). However, consistent with our observations of the EZM, we detected a significant difference between the female WT and BE5.1KO whereby WT female mice buried an average of 5 marbles each whereas female BE5.1KO animals only buried between 1 and 2 marbles within the same time frame (Fig. 7A).

### The effects of BE5.1 deletion on female anxiety-like behaviour could be reversed using diazepam

Although marble burying has been widely used as a test of anxiety there are concerns that extrapolating anxiety-like behaviour from marble burying may be compromised by the parallel manifestation of obsessive-compulsive disorder-related behaviours [34]. To better understand the contribution of anxiety-like behaviours in our results, we undertook these tests again in females after treating a subset of them with either a vehicle (saline) or saline containing 10 mg/kg^–1^ of the anxiolytic drug diazepam. As before, female WT and BE5.1KO mice treated with the vehicle displayed a significant difference in the number of marbles buried within the 10 min of the study (Fig. 7B). However, following treatment with diazepam, both groups of mice buried significantly more marbles and displayed no significant difference in the numbers of marbles buried (8–9; Fig. 7B).

### The rs10767664 polymorphism is associated with feelings of anxiety and risk taking in human cohort studies

Next, we explored whether there was evidence for a functional role of rs10767664 in anxiety-like behaviour in humans. To this end, we extracted association results for rs10767664 from all genome-wide association studies (GWAS) included in the MRC IEU OpenGWAS database [https://europepmc.org/article/ppr/ppr199104]. We restricted the GWAS results to traits related to anxiety, worry, risk taking and sexual history. In total, we extracted 51 associations (Supplementary Table S4) of which 4 relating to anxiety/worry were statistically significant after adjustment for multiple testing and found that the A-allele of rs10767664 was statistically significantly associated with anxious feelings and feelings of worry (FDR < 0.05, Table 1) consistent with the role for BE5.1 in anxiety observed with our mouse model. In addition, the analysis also emphasised a high association with higher levels of overall risk taking, earlier age at first sexual intercourse and with a higher lifetime number of sexual partners (Supplementary Table S4).

**Table 1:** Statistically significant associations of the A-allele of rs10767664 with traits related to human anxiety.

Trait	Trait ID	Sample size	Beta	SE	*P* value	FDR
Worrier/anxious feelings	ukb-b-6519	450,765	–0.005	0.001	3.30E-05	0.00056099
Worrier/anxious feelings	ukb-a-51	497,161	–0.006	0.001	9.14E-05	0.0009325
Feeling worry	GCST006950	372,869	–0.010	0.003	0.0002948	0.00250582
Worry	GCST006478	348,219	–0.010	0.003	0.0007492	0.00545849

*Beta* beta estimate for linear traits and log-odds ratio for binary traits, relative to the A-allele of rs10767664, *SE* standard error of the estimate, *FDR* false-discovery rate.

### eQTL analysis of the rs10767664 polymorphism suggest a role for BE5.1 in regulating the expression of the BDNF-AS transcript

The current study has demonstrated that the BE5.1 sequence acts as an enhancer in primary cells but that deleting BE5.1 from the mouse genome resulted in an increase in the expression of BDNF exonic mRNA in amygdala tissues in female mice. On the face of it, these observations appear to contradict each other as deleting an enhancer should not result in an increase in gene expression. For this reason, we asked whether different alleles of the rs10767664 polymorphism had an effect on the expression of genes surrounding BE5.1 (including the BDNF gene) in humans. We interrogated the GTEx database with the rs10767664 polymorphism and found a significant change in the expression of a number of genes flanking the BDNF locus including METTL15 (*p* = 0.03 in amygdala) and LIN7C (strongly affected in vascular tissue (Aorta; *p* = 4.5 × 10^–7^, tibial artery; *p* = 1.2 × 10^–4^) and less strongly in oesophagus (*p* = 1.7 × 10^–4^), muscle (*p* = 4.9 × 10^–3^) and visceral adipose tissue (*p* = 0.006). In contrast, we were unable to find data supporting a change in the expression of the BDNF gene in GTEx as a result of the rs10767664 polymorphism. Intriguingly, rs10767664 had a significant effect on the expression and splicing of the BDNF-antisense gene (BDNF-AS) gene, which expresses an alternatively spliced antisense RNA whose exon 5 sequence is complimentary to exon 9 of the BDNF mRNA transcript [35] (Table 2). This observation is especially interesting as siRNA knockdown of BDNF-AS results in an increase in both BDNF mRNA and protein [35]. The most significant effects of rs10767664 on BDNF-AS expression occurred in the nucleus accumbens; the addiction centre of the brain, and a number of cortical regions including the cingulate cortex that is a critical component of the limbic system and controls anxiety and fear (Table 2). We also observed that allelic variation at the rs10767664 polymorphism had significant effects on the splicing of the BDNF-AS primary transcript (Table 2).

**Table 2:** Effect of rs10767664 on gene expression and splicing of BDNF-AS in human brain tissue.

					Single tissue eQTL		Single tissue splicing QTL		
Tissue	Gene symbol	SNPID	EA	Samples	*p* value	NES	Intron Id [hg38]	*p* value	NES
Brain—anterior cingulate cortex (BA24)	BDNF-AS	rs10767664	A	147	**3.90E-03**	0.17	27640005:27659171:clu_4862	**7.80E-08**	–0.86
Brain—caudate (basal ganglia)	BDNF-AS	rs10767664	A	194	0.08	0.11	27640005:27659171:clu_5504	**2.10E-13**	–0.95
Brain—cerebellum	BDNF-AS	rs10767664	A	209	**0.02**	0.18	27640005:27659171:clu_6041	**1.60E-07**	–0.64
Brain—cortex	BDNF-AS	rs10767664	A	205	**8.80E-04**	0.19	27640005:27659171:clu_5702	**1.90E-09**	–0.75
Brain—frontal cortex (BA9)	BDNF-AS	rs10767664	A	175	**1.10E-04**	0.21	27640005:27659171:clu_5288	**6.20E-11**	–0.84
Brain—hippocampus	BDNF-AS	rs10767664	A	165	**0.01**	0.13	27640005:27659171:clu_5002	**3.20E-07**	–0.73
Brain—hypothalamus	BDNF-AS	rs10767664	A	170	0.07	0.09	27640005:27659171:clu_5405	**3.10E-07**	–0.73
Brain—nucleus accumbens (basal ganglia)	BDNF-AS	rs10767664	A	202	**9.70E-06**	0.22	27640005:27659171:clu_5655	**2.10E-14**	–1.00
Brain—putamen (basal ganglia)	BDNF-AS	rs10767664	A	170	**3.90E-03**	0.17	27640005:27659171:clu_4853	**3.90E-11**	–0.98
Brain—spinal cord (cervical c-1)	BDNF-AS	rs10767664	A	126	**1.70E-03**	0.21	27640005:27659171:clu_4789	**6.70E-10**	–0.95

From GTEx database.

*EA* effect allele, *SNPID* dbSNP identifier, *NES* normalized effect size (i.e., slope of the regression).

*p* < 0.05, highlighted in bold.

### QPCR analysis of BE5.2 deletion mice supports its role in modulating expression of BDNF-AS

**Fig. 8:**
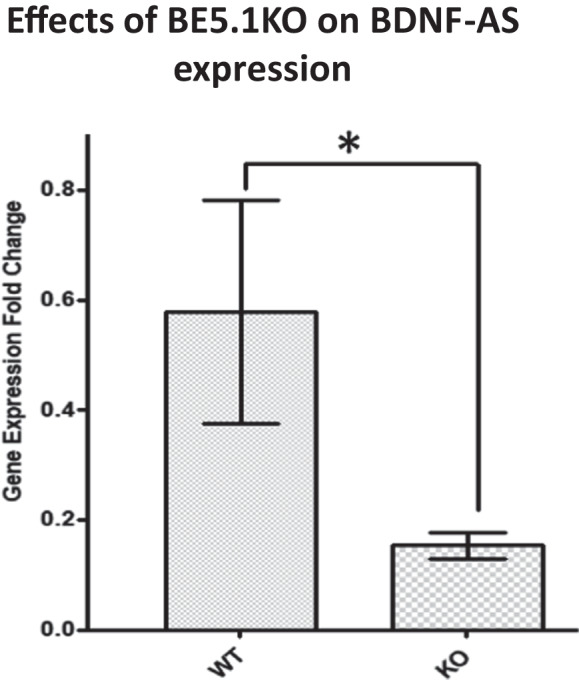
Deletion of BE5.1 reduces expression of BDNF-AS mRNA. Bar graph demonstrating the effects of deleting BE5.1 on the expression of the BDNF-AS transcript as determined using quantitative PCR on total mRNA derived from amydgala tissues from wild-type (WT) and BE5.1KO (KO) mice using primers as previously described [35]. *n* = 8; *p < 0.05; error bars = SEM.

In order to explore the possibility that BE5.1 supports the expression of BDNF-AS in mice in a manner consistent with the observed human eQTL analysis, we examined the expression of the BDNF-AS gene in the amygdala of wild-type and BE5.1KO mice using quantitative PCR as previously described [35]. We demonstrated that QPCR could detect the BDNF-AS transcript in wild-type mice but that there was a significant reduction in the detection of the BDNF-AS transcript in BE5.1KO mice (Fig. 8). This observation is consistent with the hypothesis that at least part of the role of the BE5.1 enhancer is to control BDNF expression through modulation of the BDNF-AS transcript.

## Discussion

Since the first successful GWAS was carried out in a large affected human cohort [36] it has become clear that nearly all disease-associated loci occur outside of gene coding regions [37] and many occur within presumed regulatory regions [38]. Indeed, it was further posited that ‘detailed mapping of cell-specific regulatory networks will be an essential task for fully understanding human disease biology’ [6]. However, this raises a major challenge because, compared to gene coding regions, the regulatory components that modulate these cell-specific regulatory networks are relatively unknown, so their identification and characterisation remains difficult, as does determining the effects of GWAS-identified disease-associated polymorphic variants on their activity.

The present study used comparative genomics to identify a region of deep conservation (350 million years) that we called BE5.1, within intron 3 of the BDNF gene that contained a human polymorphism highly associated with obesity [5]. Magnetofection of primary hypothalamic cells, where BDNF has been shown to modulate appetite [10], demonstrated that the T-variant of BE5.1 acted as an enhancer of BDNF promoter 4 activity but that the obesity-associated A-variant could not. In the context of the GWAS findings, this initial observation suggested that the T-allele of BE5.1, which exists within the majority of the population, promotes levels of BDNF gene expression in the hypothalamus appropriate for normal weight. These observations suggest that a contributing factor driving obesity in humans stems from a reduction in the ability of an enhancer element (BE5.1) to drive the expression of BDNF in the hypothalamus as a result of the obesity-associated A-allele. This observation was consistent with previous studies showing that deletion of BDNF in the hypothalamus led to increased food intake [28].

Based on these observations, we used injection of CAS9 mRNA and sgRNA specific for the mouse BE5.1 enhancer to produce a line of mice deficient for BE5.1. Mice homozygous for the BE5.1 deletion were viable and appeared normal. Because the rs10767664 polymorphism had been strongly associated with obesity and was shown to occur within an enhancer of BP4 activity we expected to see a significant decrease of BDNF mRNA in hypothalamic tissues, where expression of BDNF is known to affect appetite [30, 31] as well as an increase in food intake and greater weight gain in these animals. However, we did not detect any decrease in the mRNA expression of any of the five BDNF exons whose expression we analysed in the hypothalamus. Moreover, we saw no significant increase in HFD intake and no obvious change in weight gain over the 28 days of the feeding trial. Nevertheless, we did detect a marginally significant increase in CHOW diet intake suggesting that deleting BE5.1 had some effect on appetite. There are a number of possible explanations for these observations. First, it is possible that the role of the BE5.1 enhancer in appetite regulation does not reside in the hypothalamus but in a region of the brain, such as the nucleus accumbens (NAc), that regulates reward behaviour known to influence food intake [39]. This hypothesis is supported by the observation that an allelic variant of the rs10767664 polymorphism, that is strongly associated with obesity in humans, is also associated with a highly significant change of the expression of the BDNF-AS gene in the NAc (GTEx eQTL analysis) to be further discussed later. Alternatively, because we analysed BDNF in the whole hypothalamus, it is possible that the regions of the hypothalamus where BDNF expression is influenced by BE5.1 may be too small to detect using QPCR. Finer dissection of the NAc, or different nuclei within the hypothalamus, followed by QPCR may be more revealing. In addition, we have yet to determine the tissue specificity of BE5.1 using reporter transgenic mice, which we have done with other enhancers in the past [24, 25, 40–44]. Thus, although we were disappointed by the marginal nature of this result, we were satisfied that the small increase in CHOW diet intake was consistent with a possible role for BE5.1 in appetite modulation and could form the basis of a larger study. Another explanation may be the possibility that deletion of BE5.1 only manifests as obesity in the correct environment or in the presence of other permissive loci.

In contrast to our results in the hypothalamus, we found a significant increase in the expression of four BDNF mRNA isoforms in the amygdala of BE5.1KO female mice. Given the evidence above, demonstrating that BE5.1 had enhancer-like properties, the significant increase in BDNF expression in the amygdala of female BE5.1KO was not anticipated, neither was the sex specificity of this effect. A further surprise was that deletion of BE5.1 was also associated with a highly significant increase in anxiety-like behaviour in these females in both the EZM and the MBT. The role of anxiety-like behaviour in our observations of the effects of BE5.1 deletion on the MBT was confirmed using the anxiolytic drug diazepam. Although the role of BDNF in the manifestation of mood disorders such as anxiety has been well known for some time, determining whether BDNF acts as an anxiolyic or anxiogenic regulator is far from resolved [45]. For example, for many years, it was accepted that BDNF or activation of the TrkB receptor reduced anxiety and depression [46]. However, the results of other studies were consistent for a role for BDNF in increasing the pathophysiology of stress-induced anxiety [47, 48] and inhibition of the TrkB receptor has been actively explored as a potential anti-depressive treatment [45]. Superficially, the coincidental observations of increased BDNF expression in amygdala and increased anxiety-like behaviour in female mice would appear to support a role for increased BDNF expression in anxiety. However, we cannot, as yet, conclusively link one with the other and need to determine whether changes in the expression of the BDNF gene in the amygdala, or indeed some other gene in another part of the brain, are responsible. Again, this will be determined by closer dissection of the brains of BE5.1KO animals and the exploration of the effects of this deletion on the expression of other genes using next-generation technologies such as RNA-seq.

A further piece of evidence linking BE5.1 activity with anxiety came from our interrogation of the MRC IEU Open GWAS database. This analysis identified an association of the A-allele of rs10767664 with worry and anxious feelings in several large human cohorts. Again, direct functional comparisons of the effects of entirely deleting the BE5.1 enhancer against changing just 1 base pair within the enhancer are not strictly equivalent and we must temper our conclusions based on these differences. Nonetheless, if considered together; the increase in BDNF mRNA in the female amygdala, as a result of deletion of BE5.1, together with the increase in anxiety-like behaviour in female BE5.1KO mice, and the significant association of the A-allele of BE5.1 to anxiety in humans, provides a compelling case for further studies of the role of BE5.1 in anxiety. Another intriguing observation from our analysis suggests a role for the A-allele in increased risk-taking behaviours and smoking further suggesting the involvement of BE5.1 in the NAc that controls addictive behaviours.

Our interrogation of the GTex database using rs10767664 also produced an unexpected result in that allelic variation of rs10767664 had no significant effect on the expression on the BDNF gene in any of the human tissues examined by GTEx. Instead, the GTEx data suggested that rs10767664 had a significant effect on the expression of the BDNF-AS gene. BDNF-AS does not encode a protein but produces a highly regulated and spliced mRNA whose transcriptional start site lies 200 kb downstream from that of the BDNF gene and whose 5th exon is complementary to exon 9 of the BDNF gene in both mice and humans [35]. Modulation of gene expression at the transcriptional level by natural antisense (antisense encoded by the genome) has a number of different mechanisms including altering levels of DNA or histone methylation at gene promoters [49]. For example, siRNA knockdown of BDNF-AS resulted in an increase in both BDNF mRNA and a corresponding decrease in the repressive chromatin markers H3K27me3 at and EZH2 (component of the polycomb repressor complex; PRC2) at the BDNF promoter [35]. It is therefore possible that a critical component of modulating levels of BDNF mRNA essential for modulating anxiety-like behaviour in the amygdala is governed by histone methylation and polycomb binding at the BDNF promoter region governed by BE5.1 driven BDNF-AS expression. We explored this possibility by determining our ability to detect BDNF-AS transcripts in amygdala tissues derived from wild-type and knockout mice. Although BDNF-AS transcripts were not expressed at high levels in wild-type animals they were detectible. However, we were unable to detect the presence of any BDNF-AS transcripts in our BE5.1KO animals consistent with the observations made in human cohorts and with our hypothesis that BE5.1 controls the expression of the BDNF-AS transcript.

In conclusion, our study has highlighted a role for the BE5.1 enhancer in the regulation of the BDNF gene and anxiety in female mice possibly through a process that includes parallel regulation of the BDNF-AS gene. Although more work needs to be done to establish the interaction of BE5.1, BDNF and BDNF-AS, the current initial observations paint a compelling picture of the complexity of regulation of the BDNF gene in different tissues. We conclude that a major strand in the effort to understand the complexities of context-specific human BDNF gene regulation, and the influence of human disease-associated SNPs upon it, must involve a combination of comparative genomics and the use of in vivo models and behavioural testing afforded by CRISPR technologies.

### Supplementary information

S1

S2

ST1

ST2

ST3

ST4

ST5

## Data Availability

The datasets generated and/or analysed during the current study are available from the corresponding author on reasonable request.
