# Supplementary material for: An ancient polymorphic regulatory region within the BDNF gene associated with obesity modulates anxiety-like behaviour in mice and humans
Source: Mol Psychiatry. 2024 Jan 16;29(3):660–70. doi: 10.1038/s41380-023-02359-7 (PMC11153140; doi:10.1038/s41380-023-02359-7)
Supplement: Supplementary file 1 — S1 [file 41380_2023_2359_MOESM1_ESM.pptx]

## Slide 1
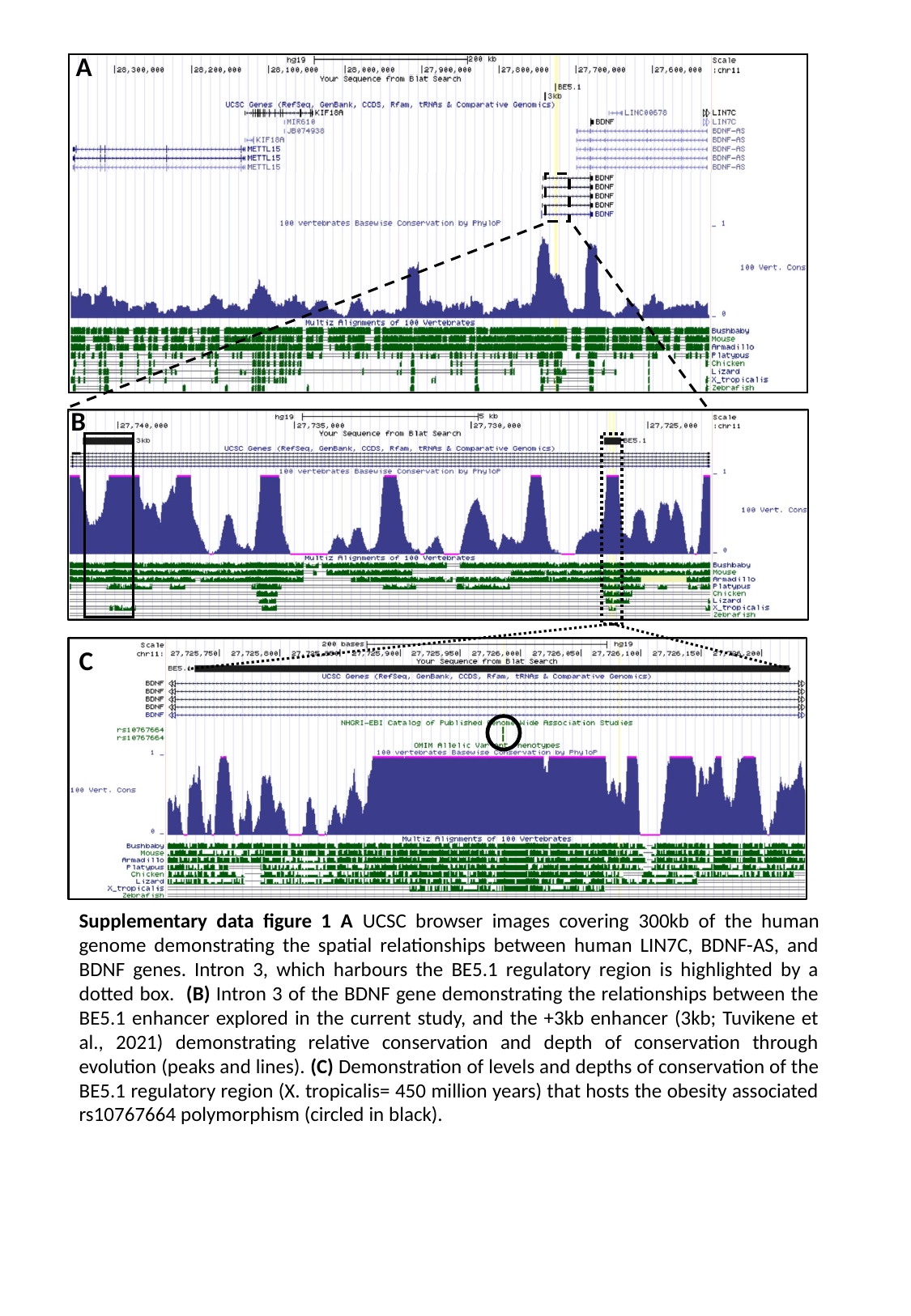

A
B
C
Supplementary data figure 1 A UCSC browser images covering 300kb of the human genome demonstrating the spatial relationships between human LIN7C, BDNF-AS, and BDNF genes. Intron 3, which harbours the BE5.1 regulatory region is highlighted by a dotted box. (B) Intron 3 of the BDNF gene demonstrating the relationships between the BE5.1 enhancer explored in the current study, and the +3kb enhancer (3kb; Tuvikene et al., 2021) demonstrating relative conservation and depth of conservation through evolution (peaks and lines). (C) Demonstration of levels and depths of conservation of the BE5.1 regulatory region (X. tropicalis= 450 million years) that hosts the obesity associated rs10767664 polymorphism (circled in black).
