# Supplementary material for: An ancient polymorphic regulatory region within the BDNF gene associated with obesity modulates anxiety-like behaviour in mice and humans
Source: Mol Psychiatry. 2024 Jan 16;29(3):660–70. doi: 10.1038/s41380-023-02359-7 (PMC11153140; doi:10.1038/s41380-023-02359-7)
Supplement: Supplementary file 2 — S2 [file 41380_2023_2359_MOESM2_ESM.docx]

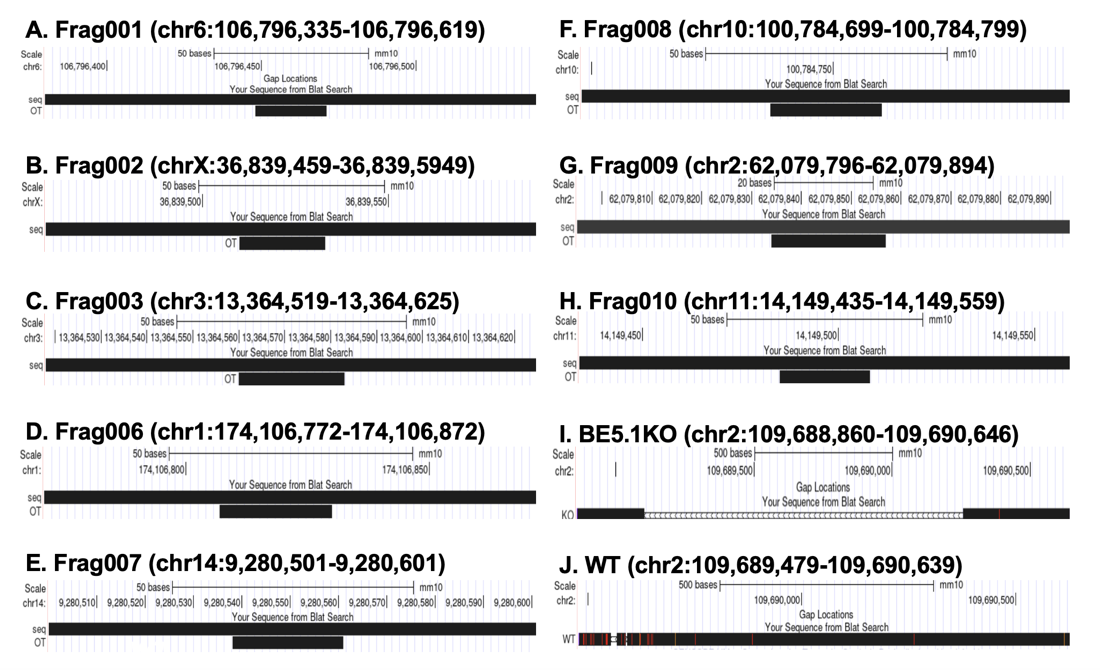


***Supplementary data figure 2*.** **UCSC genome browser BLAT comparisons of sequencing data derived from PCR products amplified from the most likely off target sites in the BE5.1KO mouse line** (*A-H*). BLAT comparisons of sequencing data (top black bar labeled “seq”) derived from PCR products amplified from BE5.1 homozygous KO earclip DNA compared to the sequence of the predicted off-target site (***Supplementary data table S1,*** bottom black bar labeled “OT”), as predicted by the CRISPOR tool addition of the ‘CRISPR Targets’ track on the UCSC genome browser. All fragments are identified by the primer combination that produced them (***Supplementary table 2***). Any insertions and deletions would be indicated by a red line in the allignment, while mismatches would be indicated by an orange line. No evidence of off target effects were observed.
