# Supplementary material for: An ancient polymorphic regulatory region within the BDNF gene associated with obesity modulates anxiety-like behaviour in mice and humans
Source: Mol Psychiatry. 2024 Jan 16;29(3):660–70. doi: 10.1038/s41380-023-02359-7 (PMC11153140; doi:10.1038/s41380-023-02359-7)
Supplement: Supplementary file 3 — ST1 [file 41380_2023_2359_MOESM3_ESM.docx]

*Supplementary Table S1*. Most likely off-target sites predicted in the mouse genome for guides (Table 1). The mismatch column displays the number of mismatches (MM) and their position (in square brackets) within the potential off-target sequence. The PAM sequence to which the gRNA:Cas9 complex could bind is italicized. AMX017 and AMX018 are represent the forward and reverse gRNAs used in McEwan *et al.* (2021) to produce BE5.1KO mice.

| **Guide** | **Off target sequence** | **Mismatches (MM)** | **Genomic Coordinates (GRCm38/mm10)** |
| --- | --- | --- | --- |
| AMX017 | 1. AGCCATACCTAAATATAGCA*TGG* 2. TGCCACATGTAAATATAACA*AGG* 3. GGTCAAATCTAAGTAAATCA*AGG* 4. GGTCAAATCTAAGTAAATCA*AGG* 5. GGTCAAATCTAAGTAAATCA*AGG* | 4MMs[1:6:8:13]  4MMs[1:9:13:18]  4MMs[3:6:16:18]  4MMs[3:6:16:18]  4MMs[3:6:16:18] | chr6:106796449- 106796471  chrX:36839511- 36839533  chr3:13364561- 13364583  chr9: 4629023- 4629045  chrX:115249118- 115249140 |
| AMX018 | 1. TTCAGTGACTTGTGCATTAT*GGG* 2. ATCAATAACCTGAGCAATAT*AGG* 3. TTTAATGTACTGTGCATTAT*AGG* 4. ATATATGGACTGTACATTAT*TGG* 5. AATAATGGCATGAGCATTAT*GGG* | 4MMs[1:5:8:10]  4MMs[7:8:13:17]  4MMs[1:3:8:9]  4MMs [3:4:9:14]  4MMs[2:3:10:13] | chr1: 174106808- 174106830  chr14: 9280539- 9280561  chr10: 100784738- 100784760  chr2: 62079835- 62079857  chr11: 14149486- 14149508 |
