# Supplementary material for: An ancient polymorphic regulatory region within the BDNF gene associated with obesity modulates anxiety-like behaviour in mice and humans
Source: Mol Psychiatry. 2024 Jan 16;29(3):660–70. doi: 10.1038/s41380-023-02359-7 (PMC11153140; doi:10.1038/s41380-023-02359-7)
Supplement: Supplementary file 4 — ST2 [file 41380_2023_2359_MOESM4_ESM.docx]

*Supplementary Table S2.* Primer sets used to amplify potential off-target sites from BE5.1KO mouse lines. The predicted amplicon length is shown alongside a numerical combination number for each primer set.

| **Off-target sequence** | **Primer Label** | **Primers (5’-3’)** | **Melting temperature (°C)** | **PCR product size (bp)** | **Primer Combination Number** |
| --- | --- | --- | --- | --- | --- |
| AMX017 #1 | xENH003 | Forward: TCCTCCATTGTTAGCATGGTCC | 60.09 | 321 | 1 |
|  | xENH004 | Reverse: GTACCCTTGATGTGCGGTGA | 60.04 |  |  |
| AMX017 #2 | xENH005 | Forward: GCGAGAAGCCGAATCAGGAG | 60.87 | 300 | 2 |
|  | xENH006 | Reverse: AGAGGCAAATGGGCAAAAGG | 62.06 |  |  |
| AMX017 #3 | xENH007 | Forward: TTATGAGGTCCCATTTGTCAATTC | 57.23 | 302 | 3 |
|  | xENH008 | Reverse: CTTGACCTTTGACAGGGGAC | 58.10 |  |  |
| AMX017 #4 | xENH009 | Forward: ACTGCCACAACTGGAGGTTAAA | 60.09 | 303 | 4 |
|  | xENH010 | Reverse: TCCCAATCTGTTGGTGGCCT | 61.43 |  |  |
| AMX017 #5 | xENH011 | Forward: TTGACAGTGTCTTTTGCCTTGC | 60.16 | 369 | 5 |
|  | xENH012 | Reverse: ACCCAGAAATGAACCCACACAT | 60.16 |  |  |
| AMX018 #1 | xENH013 | Forward: AACCCTGATCATGGCATACCT | 58.86 | 300 | 6 |
|  | xENH014 | Reverse: GCATTTGGAGATTGAATTCCGGT | 59.87 |  |  |
| AMX018 #2 | xENH015 | Forward: ACCTGGAAGAACTCAACCCC | 59.23 | 422 | 7 |
|  | xENH016 | Reverse: TGTCAATGTGGCAACAGGGTA | 59.86 |  |  |
| AMX018 #3 | xENH017 | Forward: TCCTACCACTGATGCCAGGTA | 59.99 | 366 | 8 |
|  | xENH018 | Reverse: CCTCTGAAGCATGGCGAAAC | 59.55 |  |  |
| AMX018 #4 | xENH01 | Forward: GGCTGTCATCCAGTGGTCTT | 59.67 | 317 | 9 |
|  | xENH020 | Reverse: GTGGCATTTCCTCAACTGAAGC | 60.35 |  |  |
| AMX018 #5 | xENH021 | Forward: CCACTTAGGGCCCAACCAAA | 60.18 | 323 | 10 |
|  | xENH022 | Reverse: TGGTGTCTGGAATCTAGCTCA | 58.18 |  |  |
