# Supplementary material for: An ancient polymorphic regulatory region within the BDNF gene associated with obesity modulates anxiety-like behaviour in mice and humans
Source: Mol Psychiatry. 2024 Jan 16;29(3):660–70. doi: 10.1038/s41380-023-02359-7 (PMC11153140; doi:10.1038/s41380-023-02359-7)
Supplement: Supplementary file 5 — ST3 [file 41380_2023_2359_MOESM5_ESM.docx]

| Primer name | Sequence (5’-3’) |
| --- | --- |
| mBDNFI | GTGTGACCTGAGCAGTGGGCAAAGGA |
| mBDNFII | GGAAGTGGAAGAAACCGTCTAGAGCA |
| mBDNFIII | GCTTTCTATCATCCCTCCCCGAGAGT |
| mBDNFIV | CTCTGCCTAGATCAAATGGAGCTTC |
| mBDNFIXA | CCCAAAGCTGCTAAAGCGGGAGGAAG |
| mBDNFrev: | GAAGTGTACAAGTCCGCGTCCTTA |
| TRKBFor | CTGGGGCTTATGCCTGCTG |
| TRKBRev: | AGGCTCAGTACACCAAATCCTA |

**Supplementary Table S3.** QPCR primers used to determine the effects of deleting BE5.1 on the expression of different isoforms of BDNF.
