# Supplementary material for: An ancient polymorphic regulatory region within the BDNF gene associated with obesity modulates anxiety-like behaviour in mice and humans
Source: Mol Psychiatry. 2024 Jan 16;29(3):660–70. doi: 10.1038/s41380-023-02359-7 (PMC11153140; doi:10.1038/s41380-023-02359-7)
Supplement: Supplementary file 6 — ST4 [file 41380_2023_2359_MOESM6_ESM.docx]

| **Supplementary Table S4. Association of rs10767664 with traits related to anxiety and risk** | | |  | |  | |  | |  | |
| --- | --- | --- | --- | --- | --- | --- | --- | --- | --- | --- |
|  |  |  | |  | |  | |  | |  |
| **Trait** | **Trait ID** | **Beta*** | | **SE**** | | **P-value** | | **FDR***** | |  |
| Risk taking | ukb-b-14147 | 0.00563103 | | 0.001 | | 0.000 | | 2.91E-05 | |  |
| Age first had sexual intercourse | ukb-b-6591 | -0.012893 | | 0.003 | | 0.000 | | 3.06E-05 | |  |
| Worrier / anxious feelings | ukb-b-6519 | -0.0052367 | | 0.001 | | 0.000 | | 5.61E-04 | |  |
| Risk taking | ukb-a-241 | 0.00522852 | | 0.001 | | 0.000 | | 8.05E-04 | |  |
| Worrier / anxious feelings | ukb-a-51 | -0.0058046 | | 0.001 | | 0.000 | | 9.33E-04 | |  |
| Feeling worry | ebi-a-GCST006950 | -0.0103182 | | 0.003 | | 0.000 | | 0.00250582 | |  |
| Worry | ebi-a-GCST006478 | -0.009863 | | 0.003 | | 0.001 | | 0.00545849 | |  |
| Lifetime number of sexual partners | ukb-b-4256 | 0.0063883 | | 0.002 | | 0.005 | | 0.03123776 | |  |
| Difficulty concentrating during worst period of anxiety | ukb-d-20419 | 0.0100377 | | 0.00399461 | | 0.0119815 | | 0.06789517 | |  |
| Worry too long after embarrassment | ukb-b-13653 | -0.0029388 | | 0.00128776 | | 0.0219999 | | 0.11219949 | |  |
| Infections with a predominantly sexual mode of transmission | finn-a-AB1_SEXUAL_TRANSMISSION | -0.0843 | | 0.042 | | 0.0446704 | | 0.19975085 | |  |
| Seen doctor (GP) for nerves, anxiety, tension or depression | ukb-b-6991 | -0.0023879 | | 0.00120044 | | 0.0470002 | | 0.19975085 | |  |
| Worry too long after embarrassment | ukb-a-53 | -0.0025724 | | 0.00151301 | | 0.0890984 | | 0.34953988 | |  |
| Restless during period of worst anxiety | ukb-d-20426 | 0.00768665 | | 0.00465555 | | 0.0987325 | | 0.35966839 | |  |
| Longest period spent worried or anxious | ukb-d-20420_irnt | 0.0177686 | | 0.0110783 | | 0.108748 | | 0.3697432 | |  |
| Worry too long after an embarrassing experience | ebi-a-GCST006946 | -0.0044145 | | 0.00287026 | | 0.1241 | | 0.39556875 | |  |
| Phobic anxiety disorders | finn-a-F5_PHOBANX | 0.1003 | | 0.0676 | | 0.1381 | | 0.41014737 | |  |
| Seen doctor (GP) for nerves anxiety tension or depression | ukb-a-246 | -0.0020349 | | 0.00141058 | | 0.149133 | | 0.41014737 | |  |
| All anxiety disorders | finn-a-F5_ALLANXIOUS | 0.044 | | 0.0308 | | 0.1528 | | 0.41014737 | |  |
| Frequent trouble falling or staying asleep during worst period of anxiety | ukb-d-20427 | -0.0048508 | | 0.00354244 | | 0.170901 | | 0.43579755 | |  |
| Answered sexual history questions | ukb-b-8446 | -0.0009467 | | 0.000707221 | | 0.18 | | 0.43714286 | |  |
| Generalized anxiety disorder | finn-a-F5_GAD | 0.0897 | | 0.0713 | | 0.2082 | | 0.45175375 | |  |
| Substances taken for anxiety: Medication prescribed to you (for at least two weeks) | ukb-d-20549_3 | -0.0023356 | | 0.00186109 | | 0.209488 | | 0.45175375 | |  |
| Keyed up or on edge during worst period of anxiety | ukb-d-20423 | -0.0047031 | | 0.00377307 | | 0.21259 | | 0.45175375 | |  |
| Anxiety (asthma-related co-morbidities) | finn-a-PULM_ANXIETY | 0.0384 | | 0.0339 | | 0.2571 | | 0.49382069 | |  |
| Seen a psychiatrist for nerves, anxiety, tension or depression | ukb-b-18336 | -0.0009148 | | 0.00081953 | | 0.26 | | 0.49382069 | |  |
| Lifetime number of same-sex sexual partners | ukb-b-1226 | -0.0135064 | | 0.0125386 | | 0.28 | | 0.49382069 | |  |
| Non-cancer illness code self-reported: anxiety/panic attacks | ukb-a-82 | -0.0003762 | | 0.000348268 | | 0.280095 | | 0.49382069 | |  |
| Anxiety disorders | finn-a-KRA_PSY_ANXIETY | 0.0255 | | 0.0236 | | 0.2808 | | 0.49382069 | |  |
| Non-cancer illness code, self-reported: anxiety/panic attacks | ukb-b-17243 | -0.0002862 | | 0.00029881 | | 0.34 | | 0.578 | |  |
| Substances taken for anxiety: Unprescribed medication (more than once) | ukb-d-20549_1 | -0.0007879 | | 0.000878392 | | 0.369719 | | 0.59219191 | |  |
| Mental health problems ever diagnosed by a professional: Social anxiety or social phobia | ukb-d-20544_1 | -0.0004995 | | 0.000562667 | | 0.374731 | | 0.59219191 | |  |
| Seen a psychiatrist for nerves anxiety tension or depression | ukb-a-247 | -0.0008296 | | 0.000951379 | | 0.383183 | | 0.59219191 | |  |
| Substances taken for anxiety: Drugs or alcohol (more than once) | ukb-d-20549_4 | 0.00085692 | | 0.00125374 | | 0.494299 | | 0.7414485 | |  |
| More irritable than usual during worst period of anxiety | ukb-d-20422 | 0.0025835 | | 0.00428638 | | 0.546697 | | 0.77696019 | |  |
| Other anxiety disorders | finn-a-F5_ANXIETY | 0.0215 | | 0.0372 | | 0.562999 | | 0.77696019 | |  |
| Tense, sore, or aching muscles during worst period of anxiety | ukb-d-20417 | 0.00267182 | | 0.00462738 | | 0.563677 | | 0.77696019 | |  |
| Professional informed about anxiety | ukb-d-20428 | -0.0024593 | | 0.00447465 | | 0.582594 | | 0.78067531 | |  |
| Worried most days during period of worst anxiety | ukb-d-20538 | 0.00157705 | | 0.00298263 | | 0.596987 | | 0.78067531 | |  |
| Ever worried more than most people would in similar situation | ukb-d-20425 | -0.0011961 | | 0.00239922 | | 0.61812 | | 0.78707944 | |  |
| Activities undertaken to treat anxiety: Talking therapies, such as psychotherapy, counselling, group therapy or CBT | ukb-d-20550_1 | -0.0008692 | | 0.00184643 | | 0.637834 | | 0.78707944 | |  |
| Mental health problems ever diagnosed by a professional: Anxiety, nerves or generalized anxiety disorder | ukb-d-20544_15 | -0.0007961 | | 0.00176336 | | 0.651646 | | 0.78707944 | |  |
| Number of things worried about during worst period of anxiety | ukb-d-20543 | -0.0019931 | | 0.00458248 | | 0.663616 | | 0.78707944 | |  |
| Activities undertaken to treat anxiety: Other therapeutic activities such as mindfulness, yoga or art classes | ukb-d-20550_3 | 0.00043792 | | 0.00128404 | | 0.733069 | | 0.84969361 | |  |
| Easily tired during worst period of anxiety | ukb-d-20429 | -0.0012299 | | 0.00423387 | | 0.771442 | | 0.8621672 | |  |
| Anxiety disorders | ukb-d-KRA_PSY_ANXIETY | 4.50E-05 | | 0.000159377 | | 0.777641 | | 0.8621672 | |  |
| Multiple worries during worst period of anxiety | ukb-d-20540 | -0.0007306 | | 0.0039982 | | 0.855003 | | 0.91709241 | |  |
| Victim of sexual assault | ukb-d-20531 | 0.0002993 | | 0.00177377 | | 0.866003 | | 0.91709241 | |  |
| Ever felt worried, tense, or anxious for most of a month or longer | ukb-d-20421 | -0.0003435 | | 0.00229738 | | 0.881128 | | 0.91709241 | |  |
| Stronger worrying (than other people) during period of worst anxiety | ukb-d-20542 | 0.00028398 | | 0.00416615 | | 0.945656 | | 0.96456912 | |  |
| Difficulty stopping worrying during worst period of anxiety | ukb-d-20541 | 8.28E-05 | | 0.00216873 | | 0.969553 | | 0.969553 | |  |
|  |  |  | |  | |  | |  | |  |
