# Supplementary material for: An ancient polymorphic regulatory region within the BDNF gene associated with obesity modulates anxiety-like behaviour in mice and humans
Source: Mol Psychiatry. 2024 Jan 16;29(3):660–70. doi: 10.1038/s41380-023-02359-7 (PMC11153140; doi:10.1038/s41380-023-02359-7)
Supplement: Supplementary file 7 — ST5 [file 41380_2023_2359_MOESM7_ESM.docx]

**Supplementary data 5; Health screen for the BE5.1KO mouse strain:**

| **Screening protocol** | **observation** | **Notes** |
| --- | --- | --- |
| Embryonic Lethal | No |  |
| Weaning behaviour | Normal |  |
|  |  |  |
| **Screening for visible birth defects** |  |  |
| Body size | Normal |  |
| Eye size | Normal |  |
| Eye colour | Normal |  |
| Skin colour | Normal |  |
| Skin texture | Normal |  |
| Stripes striations | No |  |
| General activity | Normal |  |
| Microagathia | No |  |
| agnathia | No |  |
| Short head | No |  |
| Scoliosis | No |  |
| Hare lip | No |  |
| Tail bend | No |  |
| Poly/syndactyly | No |  |
| Fused toes | No |  |
| Limb shape, length | No |  |
| Blebs or bruising | No |  |
| Oedema | No |  |
| hydrocephaly | No |  |
| Chylous ascites | No |  |
| Spina bifida | No |  |
|  |  |  |
| **Screening for visible pre-weaning defects** |  |  |
| Body size | Normal |  |
| Skin colour | Normal |  |
| Blotchy coat | No |  |
| Coat colour | Normal | black |
| Belly spot | No |  |
| Head blaze | No |  |
| General activity | Normal |  |
| Tremors /fits | No |  |
| Circling | No |  |
| Head weaving | No |  |
| Ataxia/ gait | No |  |
| hydrocephaly | No |  |
|  |  |  |
| **Screening for visible weaning defects** |  |  |
| Body size | Normal |  |
| Eye-size/ colour | Normal |  |
| Ear-size/ position | Normal |  |
| Coat colour/texture | Normal |  |
| Skin tension | Normal |  |
| Greasy/rough coat | No |  |
| Curly coat/ whiskers | No |  |
| Thinning/balding coat | No |  |
| Dark footpads | No |  |
| General activity | No |  |
| Tremors/fits | No |  |
| circling | No |  |
| head weaving | no |  |
| Ataxia/gait | Normal |  |
| micrognathia | No |  |
| Short/wide/ thin head | No |  |
| scoliosis | No |  |
| Tail bend | No |  |
| Poly/syndactyly | No |  |
| Fused toes | No |  |
| Limb shape, length | No |  |
| Puffy limbs/ tail | No |  |
| Belly spots | No |  |
| Head blaze | No |  |
| Coat colour | normal | Black |
| hydrocephaly | No |  |
|  |  |  |
| **5+ weeks** |  |  |
| Litter size | normal | Average 6.8 which is the same as wild type C57/BL6. |
| Litter frequency | normal |  |
| Pre wean loss | 0% |  |
| Diet and preference | N/A |  |
| Gross behavioural defects | No |  |
| Gross cognitive defects | No |  |
| Aggressive behaviours/ group housed | No |  |
|  |  |  |
